# Real World Outcomes of Patients with Aggressive Lymphoma and Autoimmune Disease Treated with CART

**DOI:** 10.3390/cancers17142358

**Published:** 2025-07-16

**Authors:** Nicole J. Altomare, Megan M. Herr, Nisha M. Nair, Deborah M. Stephens, Jonathon B. Cohen, Narendranath Epperla, Matthew Cortese, Rahul Bhansali, Tamara K. Moyo, Vaishalee Kenkre, Thomas Ollila, Brian Hess, Lindsey Fitzgerald, Geoffrey Shouse, James A. Davis, Christy Jesme, Ari Pelcovits, Jonathan Moreira, Adam Lin, Shuo Ma, Jane N. Winter, Alexey Danilov, Stefan K. Barta, Leo I. Gordon, Jason Romancik, Natalie S. Grover, Reem Karmali

**Affiliations:** 1 Feinberg School of Medicine, Northwestern University, Chicago, IL 60611, USA; nicole.altomare@northwestern.edu; 2 Roswell Park Comprehensive Cancer Center, Buffalo, NY 14203, USA; megan.herr@roswellpark.org (M.M.H.); nisha.nair@roswellpark.org (N.M.N.); matthew.cortese@roswellpark.org (M.C.); 3 Lineberger Comprehensive Cancer Center, University of North Carolina, Chapel Hill, NC 27599, USA; dmstep@email.unc.edu (D.M.S.); natalie_grover@med.unc.edu (N.S.G.); 4 Winship Cancer Institute, Emory University, Atlanta, GA 30322, USA; jonathon.cohen@emory.edu (J.B.C.); jason.t.romancik@emory.edu (J.R.); 5 James Cancer Center, Ohio State University, Columbus, OH 43210, USA; naren.epperla@hci.utah.edu; 6 Huntsman Cancer Institute, University of Utah, Salt Lake City, UT 84112, USA; lindsey.fitzgerald@hci.utah.edu (L.F.); christy.jesme@hci.utah.edu (C.J.); 7 Abramson Cancer Center, University of Pennsylvania, Philadelphia, PA 19104, USA; rahul.bhansali@pennmedicine.upenn.edu (R.B.); stefan.barta@pennmedicine.upenn.edu (S.K.B.); 8 Levine Cancer Institute, Atrium Health, Charlotte, NC 28204, USA; tamara.moyo@atriumhealth.org; 9 Carbone Cancer Center, University of Wisconsin–Madison, Madison, WI 53792, USA; vkenkre@medicine.wisc.edu; 10 Brown University Health Cancer Institute, Brown University, Providence, RI 02903, USA; thomas_ollila@brown.edu (T.O.); ari_pelcovits@brown.edu (A.P.); 11 Hollings Cancer Center, Medical University of South Carolina, Charleston, SC 29425, USA; hessbr@musc.edu (B.H.); davisjaa@musc.edu (J.A.D.); 12 City of Hope Comprehensive Cancer Center, Duarte, CA 91010, USA; gshouse@coh.org (G.S.); adanilov@coh.org (A.D.); 13 Robert H Lurie Comprehensive Cancer Center, Northwestern University, Chicago, IL 60611, USA; jonathan.moreira@nm.org (J.M.); adam.lin@nm.org (A.L.); shuo-ma@northwestern.edu (S.M.); jwinter@nm.org (J.N.W.); lgordon@nm.org (L.I.G.)

**Keywords:** Autoimmune disease, chimeric antigen receptor T-cell therapy, lymphoma

## Abstract

Background/Objectives: There is an association between autoimmune disease (AID) and non-Hodgkin lymphoma (NHL). Chimeric antigen receptor T-cell therapy (CART) is approved for the treatment of aggressive B-cell NHL (B-NHL) based on pivotal trials that excluded patients with active AID and/or on chronic immunosuppressive therapy (IST). Thus, the efficacy of CART in patients with AID warrants further investigation. Methods: We identified patients with and without AID from a cohort of 727 patients treated with CART for aggressive B-NHL across 12 institutions. Overall survival (OS) and progression-free survival (PFS) were estimated using Kaplan-Meier survival curves. AID vs. non-AID patients were compared using log-rank tests. Results: Among our patients with aggressive B-NHL, 47 (6.4%) had concurrent AID. Rates of cytokine release syndrome and neurotoxicity were not significantly different between patients with and without AID (*p* = 0.5), while infection rates were higher in the AID cohort. For AID vs. non-AID patients, complete response rates were similar between the groups (38% vs. 36.0%, *p* = 0.36). The rates of relapse/progression post-CART were not significantly different (*p* = 0.07). Three-year PFS rates were 49% and 32%, respectively (*p* = 0.18); 3-year OS rates were 57% and 44%, respectively (*p* = 0.25). Prior to CART, 32% of patients with AID were on IST; 6 (13%) patients with AID either remained on IST during CART or resumed IST post-CART, and 1 patient newly started IST following CART. Conclusions: In the real-world setting, despite inherent T-cell dysfunction expected in B-NHL patients with concurrent AID, the safety and efficacy of CART is comparable with durable responses.

## Simple Summary

Autoimmune diseases (AIDs) are associated with the development of B-cell non-Hodgkin lymphomas (B-NHLs). Autologous chimeric antigen receptor T-cell therapy (CART) is an effective therapy approved for the treatment of lymphoma; however, patients with AIDs were excluded from trials that led to CART approval. The goal of this retrospective study was to compare clinical outcomes for patients treated with CART for aggressive B-NHL with and without underlying AIDs. We found that the safety profile and efficacy of CART were comparable between these two cohorts. We also provide data on the impact of CART on AID control. This provides real-world information on the utility of CART as treatment for lymphoma in patients with AIDs, as well as insight into its possible use in the treatment of AIDs alone.

## 1. Introduction

Autoimmune diseases (AIDs) have been shown to be a risk factor for the development of non-Hodgkin lymphoma (NHL), with more than 15 million Americans diagnosed with AIDs between 2011 and 2022 [1,2,3,4,5,6,7]. The underlying mechanism for this association unclear but is thought to be related, in part, to inflammation, increased cytokines, and use of immunosuppressant medications in patients with AIDs. Given the heterogeneity of these diseases, it is likely multifactorial, and no single mechanism has emerged [2].

Pivotal prospective trials evaluating autologous CD19-directed chimeric antigen receptor T-cell therapy (CART) for the management of B-NHL have typically excluded patients with AIDs with concerns raised for the potential to harvest dysfunctional T-cells, thus impacting efficacy, or to exacerbate the underlying AID with infusion of auto-reactive T-cells [8,9,10,11]. Data addressing the safety and efficacy of CART for the treatment of lymphoma in patients with underlying AIDs is therefore limited. There is also concern that the use of immunosuppressive therapy (IST) in these patients may impact T-cell function. AIDs in clinical practice, however, are not exclusionary for the use of CART. In fact, more recently, CART has been studied for the treatment of AIDs with the realization that these diseases are often driven by B-cells. CART leads to B-cell depletion and is therefore a rational area of research in AID treatment and disease control [12,13,14,15]. As such, CART is currently being studied in a variety of rheumatologic and neurologic AIDs [12]. Our objective for this paper was to explore outcomes for patients undergoing CART for aggressive B-NHL with and without underlying AIDs. We provide real-world insight on the safety and efficacy of CART for patients with concurrent aggressive B-NHL and AID and the impact of CART on disease control for both.

## 2. Materials and Methods

We identified patients with and without AIDs from a cohort of 727 patients treated with CD19 autologous CART for aggressive B-cell NHL across 12 academic institutions in the United States between 2018 and 2024. The breakdown of lymphoma histologies is as follows: diffuse large B-cell lymphoma (547 patients), transformed follicular lymphoma (138 patients), primary mediastinal B-cell lymphoma (14 patients), Richter’s transformation (23 patients), transformed marginal zone lymphoma (24 patients), post-transplant lymphoproliferative disorder (8 patients), grey zone (2 patients), transformed mantle cell lymphoma (3 patients), Burkitt’s lymphoma (3 patients), T-cell/histiocyte-rich large B-cell lymphoma (5 patients), plasmablastic (2 patients), primary central nervous system lymphoma (1 patient), and other (23 patients). The study was approved by the respective institutional review boards and conducted in accordance with the Declaration of Helsinki.

### 2.1. Endpoints and Assessments

Cytokine release syndrome (CRS) and immune effector-cell neurologic syndrome/neurotoxicity (ICANS) were defined according to the ASTCT Consensus criteria [16]. Survival time was calculated from infusion to date of death or last follow-up. Progression was defined as the earliest of the latter or documented relapse/progression. Clinically significant infection data were collected on patients post-CART infusion. Patients with a formal diagnosis of an autoimmune condition managed with or without immunosuppression were identified, including but not limited to seropositive inflammatory arthropathy and/or vasculitis, inflammatory bowel disease, thyroid disorders, celiac disease, sarcoidosis, multiple sclerosis (MS), and myasthenia gravis (MG). Immune thrombocytopenic purpura was excluded as this was thought likely to be a result of lymphoma rather than a primary autoimmune condition. This excluded 3 patients.

### 2.2. Statistical Analyses

Data were compared using the Chi-square/Fisher’s exact tests for categorical variables and the ANOVA test for continuous variables. Overall survival (OS) and progression-free survival (PFS) were estimated using Kaplan–Meier survival curves. AIDs vs. non-AIDs patients were compared using log-rank tests. All statistical tests were performed using SAS 9.4 (Cary, NC, USA), and a *p*-value < 0.05 was considered statistically significant.

## 3. Results

Of the 727 patients, 47 patients had an underlying AID (6.4%), with baseline demographic and clinical information for these patients noted in Table 1. There was no significant difference in median age or sex between groups (*p* = 0.15 and 0.72, respectively). The most common AID was rheumatoid arthritis (RA, 29.8%), followed by systemic lupus erythematosus (SLE), Sjogren’s syndrome, Crohn’s disease, and psoriasis (10.6% each).

For AID vs. non-AID patients, there was no difference in rates of double-hit lymphoma (DHL; *p* = 0.39), double-expressor lymphoma (DEL; *p* = 0.67), primary refractory disease (*p* = 0.48), or median lines of prior therapy, including prior autologous stem cell transplant (*p* = 0.32). International prognostic index score, ECOG performance status, incidence of stage 4 disease, and elevated LDH at the time of CART collection were similar between cohorts (*p* = 0.21–1.0). After apheresis, there was no difference in the use of bridging therapy (*p* = 0.67). The median time from CART collection to infusion for non-AID and AID groups was 34 days and 36 days, respectively (*p* = 0.96). There was no difference in CART construct used (*p* = 0.07), with axicabtagene autoleucel being most commonly used in both groups.

### 3.1. Clinical Outcomes for B-NHL

**Figure 1:**
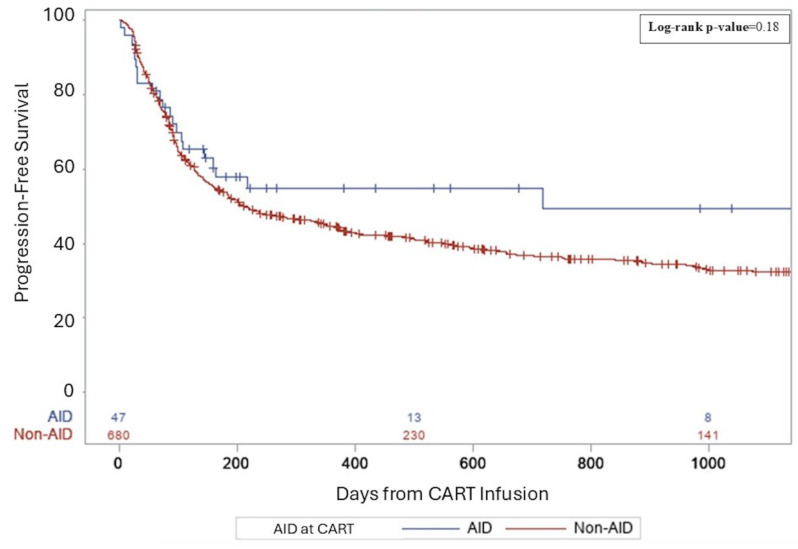
A three-year progression-free survival following CART for aggressive lymphoma.

**Figure 2:**
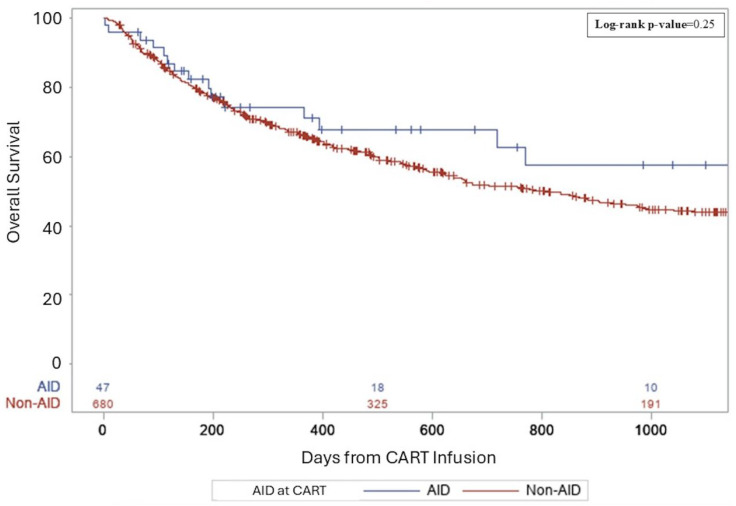
A three-year overall survival following CART for aggressive lymphoma.

Median follow-up for survivors was 26.5 months. For AID vs. non-AID groups, complete remission rates (CRs) were similar (38.3 vs. 36%, *p* = 0.36). The rates of relapse/progression post-CART were not significantly different—52.6% of the non-AID group and 38.3% in the AID group (*p* = 0.07). Three-year PFS rates from CART infusion were 49% in AID patients and 32% in non-AID patients (*p* = 0.18, Figure 1). Three-year OS rates from CART infusion were 57% in AID patients and 44% in non-AID patients (*p* = 0.25, Figure 2). There were no differences in PFS and OS between groups for primary refractory disease (*p* = 0.69 and 0.73), DHL (*p* = 0.91 and 0.61), DEL (*p* = 0.46 and 0.81), or patients requiring bridging (*p* = 0.87 and 0.7). All AIDs patients who relapsed received salvage therapy post-CART. Details regarding salvage therapies can be found in Supplemental Table S1.

### 3.2. Clinical Impact of CART on the Underlying AID

Data regarding IST resumption was available in 36 patients. Notably, 15 (31.9%) patients with AID were on immunosuppressive therapy (IST) prior to CART. Seven (14.9%) patients with AID were placed on IST following CART, only one of whom was not on IST prior to CART; three (6.4%) of these patients restarted following CART, and three (6.4%) remained on IST throughout their CART treatment process and after treatment. Of the six patients that were on IST following CART, five received IST for active AIDs and one remained on IST as prophylaxis. Four patients were documented to have flares of their AID following CART, two of which were within the first 90 days following CART. Supplemental Table S2 provides further detail on IST utilization in patients with AIDs before and after CART.

### 3.3. Toxicity

Rates of CRS were similar between non-AID and AID groups, including grade 3+ (13.2% and 12.1%, respectively, *p* = 0.5). Median time to CRS from infusion was 3 days, and duration of CRS was 4 days for both cohorts. However, tocilizumab was used more frequently in patients with AID (*p* = 0.02). Rates of ICANS were also similar between non-AID and AID groups including grade 3+ (42.4% and 40%, respectively). Time to ICANS was 6 days in both groups. Duration of symptoms was 5 days in the non-AID group and 3 days in the AID group. Persistent neutropenia (ANC < 500) and thrombocytopenia (platelets < 50) at 30 days or greater post-CART were documented in five (10.6%) and three (6.4%) AID patients, respectively, compared to 50 (7.4%) and 78 (11.4%) in non-AID (not statistically different).

Within 90 days post-CART infusion, more AID patients incurred infections. Twenty AID patients (42.6%) versus 191 non-AID patients (28.1%) had documented infections (*p* = 0.02). Four patients in the AID group experienced multiple infections during this 90-day period; the most common initial infections were bacterial (65%) and viral (35%). No patients in the AID group and eight patients in the non-AID group were diagnosed with COVID-19 as their initial infection during this time period. Two patients with AID passed away as a result of infections.

Among the 20 AID patients with infections, neutropenia post-CART was present in 16 (80%). Immunoglobulin G (IgG) levels <400 were present in 6 AID patients (30%) and 61 non-AID patients (31.9%) who developed an infection within 90 days post-CART. In the AID patients, the mean IgG level was 431.4 (range 87–646) at 30 days and 355.4 (range 115–682) at 90 days post-CART. For prophylaxis administration in AID patients, 19 (95%) were on acyclovir and 15 (75%) were on *Pneumocystis jirovecii* pneumonia (PJP) prophylaxis following CART.

## 4. Discussion

AIDs are associated with T-cell dysfunction that can be exhausted by continuous activation by self-antigen. Therefore, whether CART is just as effective in such patients for the treatment of lymphoma is an important question. In this study, we report toxicity and survival data on the largest number of patients with concurrent AID and aggressive BNHL (*de novo* diffuse large B-cell lymphoma or transformed indolent lymphoma) to date with follow-up of >2 years. We show that there are no significant differences in outcomes in patients with and without AID treated with CART for aggressive B-cell NHL. While not statistically significant, relapse rates were numerically lower in patients with AID. Despite underlying immune dysregulation expected in patients with AID, we demonstrate that the efficacy of CART does not appear to be diminished, and durable responses are feasible. This is despite previous studies illustrating that T-cells in patients with autoimmune disorders showed decreased exhaustion and cell death markers during CART manufacturing as well as in the final product. This discrepancy between our clinical results and this preclinical data may be a result of decreased signaling during CART production that leads to restoration in T-cell function [17,18].

Wang et al. found similar results in their study of matched patients with and without underlying rheumatic AID with comparable survival outcomes and toxicity rates between groups treated with CD19 CART for a heterogenous group of aggressive or indolent lymphoma. They found both AID and non-AID groups had similar times to next treatment and overall survival. They showed >50% overall survival in both groups at 1000 days post-CART, similar to our findings [17]. Aside from this data, the application of CART in patients with concurrent lymphoma and autoimmune conditions has been limited to case reports [19].

In terms of AID control post-CART, the aforementioned study also demonstrated that patients achieved better AID control of underlying rheumatic diseases, based on improvement in biochemical markers and low rates of resumption of IST. We also show low rates of flares and resumption of IST in our AID population though it was a small sample size. Though, a larger study may confirm this information and support CART use for AID control and treatment. We recognize that a limitation of our data is the lack of serologic studies that would support AID control. Unlike Wang et al., our study characterized response to CART in a more homogenous population of aggressive B-NHL. We also provided survival data in a larger variety of rheumatologic AIDs, including neurologic disorders such as multiple sclerosis, supporting CART applicability to a broader population [19]. This is particularly important when factoring in neurologic toxicities associated with CART.

These collective findings align with an emerging interest in using CART primarily for the treatment of rheumatologic and neurologic AID in patients without underlying malignancy [20,21,22,23,24]. To date, there have been case reports of CART use in a variety of AIDs, such as SLE, systemic sclerosis, RA, myasthenia gravis, and multiple sclerosis [25,26,27,28,29]. Additionally, phase I clinical trials using compound CART cells in SLE and neuromyelitis optica spectrum disorders found that this treatment was both safe and effective [30,31].

There are currently numerous clinical trials underway for the treatment of various autoimmune conditions, including SLE, MS, MG, myopathies, dermatomyositis, and others, as described in Supplemental Table S2 [12,32].

Despite the inherent limitations of a retrospective study, our data further adds to the field by providing granularity on short- and long-term toxicities of CART in patients with concurrent lymphoma and AID. Although rates of CRS and ICANS were similar, we note a higher use of tocilizumab in our AID cohort—this may have been impacted by the use of IST in the peri-CART period in 12 patients. Although rates of neutropenia were comparable between groups, the significantly higher rates of infections in the AID group following CART may also be a result of peri-CART IST. Thus, patients with AIDs undergoing CART should be managed with antibacterial prophylaxis and close monitoring of immunoglobulin levels with quick initiation of secondary prophylaxis with intravenous immunoglobulin G (IVIG) in the post-CART period.

## 5. Conclusions

In summary, despite underlying immune dysregulation expected in patients with AID, autologous CART efficacy does not appear to be diminished, and durable responses are feasible. CART should be considered a curative treatment option for patients treated for aggressive B-cell NHL despite underlying AIDs without concern for higher signals of exacerbation of the underlying AID. Biologic correlatives of T-cell function and AID status are warranted to enhance our understanding of CART impact in patients with concurrent B-NHL and AID.

## Figures and Tables

**Table 1:** Clinical and demographic features among patients at time of CART by autoimmune disease status (n = 727).

Parameter	Non AID Patients (n = 680)	%	AID Patients (n = 47)	%	*p*-Value
Median age at diagnosis, years (range)	58	(17–85)	61	(28–83)	0.15
Sex					0.72
Female	243	35.7	18	38.3	
Male	437	64.3	29	61.7	
Race					0.03
White	572	84.1	45	95.7	
Non-White/unknown	108	15.9	2	4.3	
Ethnicity					0.11
Non-Hispanic	628	92.4	46	97.9	
Hispanic	44	6.5	0	0.0	
Missing	8	1.2	1	2.1	
ECOG at dx					0.18
0–1	498	73.2	29	61.7	
2+	50	7.4	4	8.5	
Missing	132	19.4	14	29.8	
IPI at dx					0.22
0–1	116	17.1	4	8.5	
2	118	17.4	13	27.7	
3	157	23.1	8	17.0	
4–5	86	12.6	5	10.6	
Missing	203	29.9	17	36.2	
Autoimmune condition					< 0.01
None	680	100.0	0	0.0	
RA	0	0.0	14	29.8	
SLE	0	0.0	5	10.6	
Sjogren’s	0	0.0	5	10.6	
Crohn’s	0	0.0	5	10.6	
Psoriasis/Psoriatic/Arthritis	0	0.0	5	10.6	
Other	0	0.0	13	27.7	
Primary refractory disease with frontline therapy					0.48
PD with frontline therapy	97	14.3	3	6.4	
Relapse within 6 months	169	24.9	10	21.3	
Relapse within 12 months	139	20.4	10	21.3	
DEL					0.39
No	339	49.9	22	46.8	
Yes	146	21.5	14	29.8	
Missing	195	28.7	11	23.4	
DHL or THL					0.67
No	437	64.3	28	59.6	
Yes	106	15.6	7	14.9	
Missing	137	20.1	12	25.5	
Bridging prior to CART					0.61
No	5	0.7	0	0.0	
Yes	329	48.4	20	42.6	
Missing	346	50.9	27	57.4	
Frontline therapy					0.32
R-CHOP	418	61.5	26	55.3	
DA-R-EPOCH	122	17.9	7	14.9	
R-ICE **	12	1.8	0	0.0	
DHAP **	2	0.3	1	2.1	
Hyper-CVAD	8	1.2	1	2.1	
R-ESHAP **	2	0.3	0	0.0	
R-bendamustine	21	3.1	1	2.1	
Other	47	6.9	4	8.5	
Missing	48	7.1	7	14.9	
Median lines of prior therapy, (range)	2	(1–8)	2	(1–8)	0.73
Missing	4	0.6	0	0.0	
Autologous transplant prior to CART					0.93
No	546	80.3	38	80.9	
Yes	134	19.7	9	19.1	

Chi-square or Fisher’s exact test for categorical variables and ANOVA test for continuous variables. ** The patients that received these therapies were predominantly indolent lymphoma patients who received R-CHOP for their indolent disease and then transformed after ≥ 1 line of therapy.

## Data Availability

The raw data supporting the conclusions of this article will be made available by the authors upon request.

## Supplementary Materials

The following supporting information can be downloaded at https://www.mdpi.com/article/10.3390/cancers17142358/s1: Table S1: Post-CART salvage regimens; Table S2: AID conditions and IST resumption; Table S3: Current clinical trials investigating the use of CART in AID.

## Abbreviations

The following abbreviations are used in this manuscript.

AID	Autoimmune disease
B-NHL	B-cell non-Hodgkin lymphoma
CART	Autologous chimeric antigen receptor T-cell therapy
IST	Immunosuppressive therapy
SLE	Systemic lupus erythematous
RA	Rheumatoid arthritis
MS	Multiple sclerosis
MG	Myasthenia gravis
ICANS	Immune effector cell-associated neurotoxicity syndrome
CRS	Cytokine release syndrome
IVIG	intravenous immunoglobulin G
OS	Overall survival
PFS	Progression free survival
