# Supplementary material for: Real World Outcomes of Patients with Aggressive Lymphoma and Autoimmune Disease Treated with CART
Source: Cancers (Basel). 2025 Jul 16;17(14):2358. doi: 10.3390/cancers17142358 (PMC12294016; doi:10.3390/cancers17142358)
Supplement: Supplementary file 1 [file cancers-17-02358-s001.zip › cancers-3696660-supplementary materials.pdf]

cancers

MDPI

Supplemental Materials

Table S1. Post-CART salvage regimens

	Post CART Regimen 1	Post CART Regimen 2	allo consolidation after cart	Treated with BiSpecific
1	-	-	-	-
2	-	-	-	-
3	-	-	-	-
4	R-polatuzumab/bendamustine	Revlimid + tafasitamab	-	-
5	R-bendamustine	-	-	-
6	-	-	Yes	-
7	XRT	-	-	-
8	-	-	-	-
9	-	-	-	-
10	-	-	-	-
11	Rituxan/gemcitabine/oxaliplatin	TAK-007-2001*	Yes	Yes
12	-	-	-	-
13	Epcortimab + zanubrutinib + dex	-	-	Yes
14	-	-	-	-
15	-	-	-	-
16	Polatuzumab + Obinutuzumab/Ritux	MUD HSCT	Yes	-
17	-	-	-	-
18	-	-	-	-
19	XRT x5 fractions	-	-	Yes
20	-	-	-	-
21	-	-	-	-
22	-	-	-	-
23	Etoposide	-	-	-
24	-	-	-	-
25	Tafasitimab + Lenalinomide	-	-	-
26	-	-	-	-
27	Tafasitimab + Revlimid	-	-	-
28	Loncastuximab tesirine	Epcoritamab	-	Yes
29	-	-	-	-
30	-	-	-	-
31	Tafasitimab + Lenalinomide	-	Yes	-
32	-	-	-	-
33	acalabrutinib	-	-	-
34	-	-	-	-
35	WBRT	-	-	-
36	XmAb1367**	ibrutinib	-	Yes
37	-	-	-	-
38	-	-	-	-
39	-	ibrutinib	-	-
40	-	-	-	-
41	R-ICE	Auto	-	-
42	R-GemOx	-	-	-
43	-	-	-	-
44	-	-	-	-
45	-	-	-	-
46	-	-	-	-

47	-	`	-	-

*CD19 CAR-NK therapy. **Plamotamab, anti-CD3/CD20 antibody.

Table S2: AID conditions and IST resumption

	AID	IST agent prior to CART	IST resumed?	IST agent	AID flare at 3 months post CART	AID flare at 6 months post CART	AID flare at 12 months post CART	AID flare at 24 months post CART
1	RA	-	-	-	No	No	No	No
2	RA	-	-	-	No	No	No	No
3	Ankylosing spondylitis	-	-	-	No	N/A	N/A	N/A
4	Psoriasis	-	-	-	No	No	No	No
5	Grave's disease	-	-	-	No	N/A	N/A	N/A
6	Hashimoto's thyroiditis	-	-	-	No	No	No	No
7	Crohn's Disease	-	-	-	No	No	No	No
8	Autoimmune hemolytic anemia	Prednisone	Yes	Prednisone	No	No	No	No
9	Celiac disease	-	-	-	No	No	N/A	N/A
10	Crohn's Disease	-	-	-	No	No	No	No
11	Crohn's Disease	-	-	-	No	No	N/A	N/A
12	Sarcoidosis	-	-	-	No	NYR	NYR	NYR
13	Sjogren's	Hydroxychloroquine	Never stopped	Hydroxychloroquine	No	N/A	N/A	N/A
14	Psoriasis	Secukinumab	-	-	No	No	No	No
15	Psoriasis	Guselkumab	Yes	Apremilast	No	No	Yes	NYR
16	Psoriasis	-	-	-	No	No	No	NYR
17	RA	Methotrexate	-	-	No	No	No	NYR
18	RA	Adalimumab	-	-	No	No	No	NYR
19	Sjogren's, Primary biliary cholangitis, cutaneous vasculitis	Prednisone	Never stopped	Prednisone	No	Yes	N/A	N/A
20	Celiac disease	-	-	-	No	No	No	NYR
21	Goodpasture's	Tacrolimus, steroids	Never stopped	Tacrolimus, steroids	N/A	N/A	N/A	N/A
22	Sjogren's	-	-	-	No	No	No	No
23	Lupus and scleroderma	Methotrexate	-	-	No	No	N/A	N/A
24	SLE	-	-	Triamcinolone, hydroxychloroquine	Yes	Yes	Yes	No
25	RA	Methotrexate	-	-	No	No	N/A	N/A
26	Sarcoidosis	-	-	-	No	No	No	NYR
27	Ankylosing spondylitis	-	-	-	No	N/A	N/A	N/A
28	RA	-	-	-	No	No	No	No
29	RA	Methotrexate and prednisone	-	-	Yes	Yes	No	No

30	Sjogren's	-	-	-	No	No	No	No
31	RA	-	-	-	NC	NC	NC	NC
32	RA	-	-	-	NC	NC	NC	NC
33	RA	-	-	-	NC	NC	NC	NC
34	RA	-		-	NC	NC	NC	NC
35	Crohns	-	-	-	NC	NC	NC	NC
36	Myasthenia gravis	-	-	-	NC	NC	NC	NC
37	SLE	Mycopheno-late and hy-droxychloro-quine	-	-	No	No	No	No
38	SLE	-	-	-	NC	No	No	No
39	RA	-	-	-	NC	NC	NC	NC
40	Autoimmune colitis from Pembro (prior to CAR-T)	-	-	-	NC	NC	NC	NC
41	SLE	-	-	-	NC	NC	NC	NC
42	RA	-	-	-	NC	NC	NC	NC
43	Sjogrens	Hy-droxychloro-quine	Yes	Hy-droxychloro-quine	No	No	No	No
44	RA	-	-	-	No	No	No	No
45	Multiple sclero-sis	-	-	-	No	No	No	N/A
46	Crohn's Disease	Infliximab --> Vedolizumab (stopped at time of lym-phoma diag-nosis)	-	-	No	No	No	N/A
47	Psoriasis	Apremilast	-	-	No	No	No	No

Descriptions of IST regimens prior and post CART and AIF flares. NYR – Not yet reached. N/A patient passed before time reached. NC - Not collected.

Table S3: Current clinical trials investigating the use of CART in AID

NCT number	AID	Target
NCT05239702	Crohn's disease<br>Ulcerative Colitis<br>Dermatomyositis<br>Still disease	CD7
NCT06154252	idiopathic Inflammatory Myo-pathy, dermatomyositis, Anti-Synthetase-Syndrome, Immune-Mediated Necrotizing Myopa-thy	CD19
NCT05085418	Immune nephritis	CD19/BMCA
NCT05085418	Immune nephritis	CD19/BMCA
NCT05938725	Lupus nephritis	CD19
NCT05798117	Lupus nephritis	CD19
NCT05828225	MG	CD19
NCT04146051	MG	BCMA
NCT06220201	MS	CD19
NCT06138132	MS	CD19

NCT06193889	MS	CD19
NCT04561557	Neuromyelitis optica spectrum disorder, MG, Chronic inflammatory demyelinating, Immune-mediated necrotising myopathy Polyradiculoneuropathy	BMCA
NCT05263817	Refractory POEMS syndrome Amyloidosis Autoimmune haemolytic anaemia Vasculitis	CD19/BMCA
NCT05085431	Sjogren's syndrome	CD19/BMCA
NCT05869955	SLE	CD19
NCT05765006	SLE	CD19
NCT03030976	SLE	CD19
NCT06189157	SLE	CD19
NCT06121297	SLE	CD19
NCT06106906	SLE	CD19
NCT05474885	SLE	CD19/BMCA
NCT05846347	SLE	CD19/BMCA
NCT06153095	SLE	CD19/CD29
NCT05930314	SLE	CD19
NCT05858684	SLE	CD19/BMCA
NCT05030779	SLE	CD19/BMCA
NCT05085444	Sleroderma	CD19/BMCA
NCT05859997	Systemic lupus erythematosus, Sjogren's syndrome, Diffuse scleroderma, Inflammatory myopathy, ANCA-associated systemic vasculitis, Antiphospholipid syndrome	CD19
